# Elevated heterotrophic capacity as a strategy for Mediterranean corals to cope with low pH at CO_2_ vents

**DOI:** 10.1371/journal.pone.0306725

**Published:** 2024-07-30

**Authors:** Ann Marie Hulver, Chloé Carbonne, Nuria Teixidó, Steeve Comeau, Dustin W. Kemp, Elise F. Keister, Jean-Pierre Gattuso, Andréa G. Grottoli

**Affiliations:** 1 School of Earth Sciences, The Ohio State University, Columbus, Ohio, United States of America; 2 CNRS-INSU, Laboratoire d’Océanographie de Villefranche, Sorbonne Université, Villefranche-sur-Mer, France; 3 Department of Integrated Marine Ecology, Stazione Zoologica Anton Dohrn, Ischia Marine Center, Ischia, Naples, Italy; 4 Department of Biology, The University of Alabama at Birmingham, Birmingham, Alabama, United States of America; 5 Institute for Sustainable Development and International Relations, Sciences Po, Paris, France; Helmholtz-Zentrum fur Ozeanforschung Kiel, GERMANY

## Abstract

The global increase in anthropogenic CO_2_ is leading to ocean warming and acidification, which is threatening corals. In Ischia, Italy, two species of Mediterranean scleractinian corals–the symbiotic *Cladocora caespitosa* and the asymbiotic *Astroides calycularis*–were collected from ambient pH sites (average pH_T_ = 8.05) and adjacent CO_2_ vent sites (average pH_T_ = 7.8) to evaluate their response to ocean acidification. Coral colonies from both sites were reared in a laboratory setting for six months at present day pH (pH_T_ ~ 8.08) or low pH (pH_T_ ~7.72). Previous work showed that these corals were tolerant of low pH and maintained positive calcification rates throughout the experiment. We hypothesized that these corals cope with low pH by increasing their heterotrophic capacity (i.e., feeding and/or proportion of heterotrophically derived compounds incorporated in their tissues), irrespective of site of origin, which was quantified indirectly by measuring δ^13^C, δ^15^N, and sterols. To further characterize coral health, we quantified energy reserves by measuring biomass, total lipids, and lipid classes. Additional analysis for *C*. *caespitosa* included carbohydrates (an energy reserve) and chlorophyll *a* (an indicator of photosynthetic capacity). Isotopic evidence shows that ambient-sourced Mediterranean corals, of both species, decreased heterotrophy in response to six months of low pH. Despite maintaining energy reserves, lower net photosynthesis (*C*. *caespitosa)* and a trend of declining calcification (*A*. *calycularis*) suggest a long-term cost to low heterotrophy under ocean acidification conditions. Conversely, vent-sourced corals maintained moderate (*C*. *caespitosa*) or high (*A*. *calycularis*) heterotrophic capacity and increased photosynthesis rates (*C*. *caespitosa*) in response to six months at low pH, allowing them to sustain themselves physiologically. Provided there is sufficient zooplankton and/or organic matter to meet their heterotrophic needs, vent-sourced corals are more likely to persist this century and potentially be a source for new corals in the Mediterranean.

## Introduction

Anthropogenic CO_2_ emissions, primarily from the burning of fossil fuels, have caused an unprecedented increase of atmospheric CO_2_ since the industrial revolution [1]. In the decade 2012–2021, 26% of total CO_2_ emissions were absorbed into the oceans [2]. When the ocean absorbs CO_2_ it disrupts the carbonate equilibrium via the production of hydrogen and bicarbonate ions, coupled with a decrease in carbonate ions and aragonite saturation state [3, 4] (Ω) resulting in a net decrease in seawater pH referred to as ocean acidification (OA). By the end of this century, under SSP5-8.5 the pH of the surface ocean is expected to decrease by 0.39 relative to 2006–2015 if emissions are not curbed [5]. Calcifying organisms such as corals can be greatly affected by OA due to their dependance on carbonate for their aragonitic skeletons [4, 6] and the increased energetic demand of calcification at low pH [7–9]. However, not all calcifying organisms are negatively affected by OA and some are able to maintain important calcification rates in environments with persistently low pH [10], possibly due to increased concentrations of bicarbonate [11]. Coral reefs in the tropics and solitary coral colonies in the Mediterranean Sea are critical to the healthy functioning of ecosystems that support marine life [12–15]. Changes in coral calcification and growth due to declining pH could destabilize these ecosystems.

Natural marine volcanic vent systems release CO_2_ into the water column, locally acidifying the seawater to pH levels expected later this century [16]. At CO_2_ vents in Papua New Guinea and Ischia, Italy, species richness, habitat complexity, and functional diversity decrease with decreasing pH along natural gradients [17–19], with calcifying species being most affected [16–20]. In Ischia, two species of coral, the symbiotic *Cladocora caespitosa* and the asymbiotic *Astroides calycularis*, have recently been found growing near CO_2_ vents [18, 21]. Although colony size is smaller at vent sites compared to nearby ambient sites due to increased bioerosion [10, 18, 21], these colonies continue to grow, suggesting local adaptation or acclimatization in response to low pH. Recent work shows that *C*. *caespitosa* and *A*. *calycularis* collected from both CO_2_ vent and adjacent ambient sites maintain calcification for up to six months when experimentally exposed to pH_T_ values as low as 7.7 in a laboratory setting [21], indicating a tolerance for a wide range of pH conditions. It is unlikely that decreases in coral energy expenditure (i.e., metabolism) contribute to maintenance of calcification. Approximately 80% of the carbon used in calcification is derived from CO_2_ respired by the coral host [22], and therefore decreased host metabolism would result in lower concentrations of metabolic CO_2_ and decreased calcification [23]. Nitrogen also appears to be essential for coral calcification [24, 25], which is primarily derived through coral heterotrophy [26]. Additionally, calcification in symbiotic corals increases with higher photosynthetic rates [27]. However *C*. *caespitosa* had similar photosynthesis rates in all experimental treatments [21], suggesting another mechanism for sustaining calcification at low pH. One potential pathway for supporting calcification at low pH is elevated heterotrophy which could provide the necessary energy to support calcification.

Heterotrophic acquisition of zooplankton, dissolved organic matter (DOM), and particulate organic matter (POM) are critical food sources for corals and provide them with the needed fixed carbon for tissue growth as well as synthesis and maintenance of fat reserves [26, 28–30]. Some species of corals are heterotrophically plastic and can increase their uptake of heterotrophic carbon in response to heat stress [31–34] and low pH [35]. Such heterotrophic plasticity enables corals at lower pH to maintain growth rates [35]. In addition, some tropical coral species have high baseline feeding capacity, providing them with sufficient nutritional stability to impart resilience following heat-stress events [33, 36, 37]. While most of these studies have been conducted on tropical corals, Mediterranean corals are more dependent on heterotrophy than their tropical counterparts [38, 39]. Even the temperate symbiotic coral *C*. *caespitosa* has higher baseline heterotrophy than tropical corals and is heterotrophically plastic in response to changes in light [40]. Therefore, the ability of Mediterranean corals *C*. *caespitosa* and *A*. *calycularis* to cope with lower pH might be facilitated by their capacity for heterotrophic plasticity and/or high heterotrophic capacity, which could lead to increased tissue thickness, energy reserves, photosynthesis, and calcification [26, 30, 41].

In this study, coral colonies of *C*. *caespitosa* and *A*. *calycularis* collected from ambient pH sites and natural CO_2_ vent sites were exposed to present day (pH_T_ 8.08) and low pH conditions (pH_T_ 7.72) during a 6-month laboratory experiment [21]. We measured a suite of physiological and biogeochemical variables including biomass, total lipids, lipid classes (phospholipids, sterols, triacylglycerols, and wax esters), total carbohydrates and chlorophyll *a* (*C*. *caespitosa* only), δ^13^C/ δ^15^N of coral tissue, and δ^13^C/δ^15^N of algal endosymbionts (*C*. *caespitosa* only), to evaluate the response of coral heterotrophic capacity and physiology to the treatments. Heterotrophic capacity is the amount of zooplankton and/or particulate organic matter corals can ingest. Here, we used isotope and lipid classes as a measure of the contribution of heterotrophic sources to coral tissues, which are proxies for heterotrophic capacity [42–44]. Coral tissue δ^13^C decreases and δ^15^N increases as the incorporation of heterotrophic nutrition to the coral tissues increases. Biomass, total lipids, and carbohydrates are measures of coral energy reserves [45], and chlorophyll *a* is a measure of stress in symbiotic corals [46]. With these measurements, we tested the hypothesis that coral colonies increase their heterotrophic capacity (i.e., feeding and/or proportion of heterotrophically derived compounds incorporated in their tissues) in response to low pH, irrespective of their site of origin. We also evaluated the corresponding physiological responses of the corals to low pH. Our findings from this study demonstrate the importance of heterotrophy in coral persistence under ocean acidification conditions.

## Methods

### Study sites

*C*. *caespitosa* and *A*. *calycularis* are temperate scleractinian corals found in the Mediterranean Sea. *C*. *caespitosa* is symbiotic with the endosymbiotic algae *Breviolum psygmophilum* and *Philozoon sp*. depending on location (S1 Table in S1 File) [47–51]. *A*. *calycularis* is not symbiotic with endosymbiotic algae and considered purely heterotrophic. Populations of *C*. *caespitosa* and *A*. *calycularis* naturally occur at distinct CO_2_ vent sites and nearby ambient pH sites around the island of Ischia, Italy (S1 Fig in S1 File). At the study sites, solitary colonies of *C*. *caespitosa* occur at open rocky reef sites at approximately 10 meters depth, whereas *A*. *calycularis* is found in 1–5 meters depth under overhangs or in shallow caves. *C*. *caespitosa* colonies were collected from the ambient site Chiane del Lume Ambient (40.718°N, 13.965°E, average pH_T_ of 8.05) and the adjacent CO_2_ vent site Chiane del Lume Vent (40.718°N, 13.960°E, average pH_T_ of 7.91) 100 meters away [21]. *A*. *calycularis* colonies were collected from the ambient site San Pancrazio (40.701°N, 13.954°E, average pH_T_ of 8.05) and the nearby CO_2_ vent site Grotta del Mago 1.5 km away (40.712°N, 13.964°E, average pH_T_ of 7.88) [10]. At both vent sites, gas released is 93% CO_2_ with undetectable levels of hydrogen sulfide [10, 21].

### Experimental set-up

Ramets of individual colonies of *C*. *caespitosa* and *A*. *calycularis* were collected at ambient (n = 16 and 14, respectively) and CO_2_ vent (n = 14 and 27, respectively) sites on May 7–8, 2019 and brought back to Laboratoire Océanographique de Villefranche, France, where a manipulative tank experiment was conducted from 15 May– 2 December 2019. Full details of the experimental set-up are provided in Carbonne et al. [21] and S1 Table in S1 File. In brief, half of the colonies of each species and site of origin (ambient vs vent) were placed in one of 12 flow-through independent tanks that received ambient pH seawater (Present day pH treatment, pH_T_ = 8.08 ± 0.01), and the other half of the colonies were placed in 12 flow-through tanks that received low pH seawater (Low pH treatment, pH_T_ = 7.72 ± 0.01). Seawater pH was lowered by bubbling CO_2_ gas. Low pH values correspond to predicted end of the century pH under SSP5-8.5 CO_2_ emissions [5]. Seawater was pumped in from 5m depth in Villefranche Bay to maintain continuous flow and ambient temperature. *C*. *caespitosa* colonies were maintained on a diurnal light cycle with maximum irradiance values of 180 μmol photons m^-2^ s^-1^ similar to irradiance levels at 10m depth where colonies were collected, which follows best practices [46]. The tops of experimental tanks containing *A*. *calycularis* colonies were covered with dark plastic bags to shade colonies from direct light and to mimic the light conditions from their cave collection site. All coral were fed freshly hatched *Artemia* nauplii three times a week, which is sufficient for corals in moderate and long-term experiments [46]. Coral calcification, respiration, and photosynthesis were measured at the end of the study, and the results are reported in Carbonne et al [21]. A subset of the experimental fragments were then frozen at -80°C and sent to the Ohio State University for additional physiological and isotopic analyses.

### Sample preparation

Coral ramets were photographed from all four sides and the top of the colony against a standard white background with a ruler for scale. Total surface area was determined using ImageJ [52] and total number of polyps counted. All non-coral tissue (i.e., filamentous algae, encrusting organisms, and boring organisms) was removed from the coral inter-polyp surface. Coral polyps were cut away from the rest of the coral skeleton using a Dremel power tool with a diamond tip, homogenized using a mortar and pestle, and partitioned into four 0.5g subsamples for physiological analyses and an additional 0.5g subsample for isotopic analyses.

### Physiological analyses

Photosynthesis of *C*. *caespitosa* was measured as the production of O_2_ during 30–60 minute incubations in 500-mL transparent chambers under irradiance of 180 μmol photons m^2^ s^-1^ using a fiber optic oxygen sensor (PreSens, OXY-4 mini) according to methods in Carbonne et al. [20]. Total chlorophyll *a* was extracted using 100% acetone [53] and standardized to ash-free dry weight (AFDW). Respiration was measured in darkness during a 30–60 minute incubation [20]. Calcification was measured using the buoyant weight technique [54] between the start and end of the experiment (202 d) [20]. Tissue biomass was calculated by determining the AFDW of a sample with known surface area [55]. Total lipids were extracted using chloroform:methanol (2:1, v:v) for *C*. *caespitosa* [56] and 100% chloroform for *A*. *calycularis* (modified from [56]). Total lipid extraction was modified for *A*. *calycularis* to prevent extraction of the secondary orange pigment that is abundant in this species and is soluble in methanol. Carbohydrates were measured using the phenol-sulfuric acid method [57]. Total lipids and carbohydrates were converted to Joules [58] and standardized to AFDW. The lipid classes of phospholipids, sterols, triacylglycerols, and wax esters were analyzed with an Iatroscan MK 6s thin layer flame ionization detector according to Keister et al. [59] and standardized to AFDW.

### Stable isotope and heterotrophic capacity analyses

Stable carbon (δ^13^C) and nitrogen (δ^15^N) isotopic analyses were conducted on animal host and algal endosymbiont fractions of *C*. *caespitosa* and whole tissue of *A*. *calycularis*. Ground coral sample was used to ensure all tissue was represented. The coral tissue was separated from the skeleton following methods from McLachlan et al. [60]. Whole *C*. *caespitosa* tissue was separated into host and algal endosymbiont fractions following methods from Price et al. [61]. Whole *A*. *calycularis* tissue was homogenized, sonicated, then dried under argon gas.

All samples were sent to University of California Davis Stable Isotope Facility for isotopic analysis. Tissue samples were combusted using a PDZ Europa 20–20 isotope ratio mass spectrometer (IRMS). The carbon isotopic values of the animal host (δ^13^C_h_), algal endosymbiont (δ^13^C_e_), and whole tissue (δ^13^C_w_) are reported as the per mil deviation of the ratio of the stable isotopes ^13^C:^12^C relative to Vienna-Peedee Belemnite Limestone Standard (v-PDB). Repeated measures of an internal standard had a standard deviation of ± 0.09 ‰ for δ^13^C. The nitrogen isotopic values of the animal host (δ^13^N_h_), algal endosymbiont (δ^13^N_e_), and whole tissue (δ^13^N_w_) are reported as per mil deviation of the ratio of the stable isotopes ^15^N:^14^N relative to air. Repeated measures of an internal standard had a standard deviation of ± 0.09 ‰ for δ^15^N. At least 10% of all measurements were made in duplicate and the SD of these measurements was ± 0.19‰ and ± 0.77‰ for *C*. *caespitosa* δ^13^C and *A*. *calycularis* δ^13^C respectively, and ± 0.16 ‰ for all δ^15^N measurements. Due to the high variability in *A*. *calycularis* δ^13^C duplicates, this data was not used in analyses. Additionally, samples with deviation ±2 SD which also had sample sizes less than the smallest reference were removed from analysis.

The difference between δ^13^C_h_ and δ^13^C_e_ (δ^13^C_h-e_) was computed to assess the proportionate contribution of heterotrophically and photoautrophically acquired carbon contribution to the coral tissue [42, 44, 62] of the symbiotic coral *C*. *caespitosa*. Lower δ^13^C_h-e_ values indicate that heterotrophy contributes relatively more carbon to coral tissues than photosynthesis, and vice versa. Unfortunately, coral δ^13^C values in the ocean acidification treatment were contaminated from the isotopically depleted CO_2_ gas used to lower the seawater pH in the tanks, confounding the biological δ^13^C signature. Therefore, δ^13^C isotopes were only interpreted in the corals from the present day pH treatment. The difference between δ^15^N_h_ and δ^15^N_e_ (δ^15^N_h-e_) was computed as a second indicator of the proportionate contribution of heterotrophy and photoautotrophy to the coral tissue where higher values indicate that heterotrophy contributes relatively more nitrogen to coral tissues than photoautotrophy, and vice versa [44]. For the asymbiotic coral *A*. *calycularis* in this experiment, changes in δ^13^C_w_ and δ^13^N_w_ should primarily be due to differences in heterotrophic food sources.

SIBER (Stable Isotope Bayesian Ellipses in R; [63]) analysis with maximum likelihood ellipses encompassing 40% and 95% of the variation were fitted to isotopic values of host and algal endosymbiont tissue of *C*. *caespitosa* from present day pH treatments to determine trophic strategy of these symbiotic corals. Percent overlap of the host and endosymbiont standard ellipse areas corrected for sample size (SEAc) as a proportion of the host SEAc was calculated to determine trophic strategy [64]. Lower overlap indicates lower amounts of resource sharing and higher heterotrophic contributions. SIBER analysis was not possible for *A*. *calycularis* as it is asymbiotic.

### Statistical analysis

Multivariate statistical analyses were conducted to determine if physiological profiles (biomass, lipids, lipid classes, calcification, and respiration) of the two species differed. Additionally, multivariate statistical analyses were conducted on each species separately to evaluate how their physiological profiles (i.e., biomass, lipids, lipid classes, calcification, respiration, photosynthesis, and δ^15^N) varied among sites of origin and treatment. Differences in physiological profiles were evaluated using two-way PERMANOVA analyses where site of origin and treatment were fixed effects, and the data was visualized using non-metric multidimensional scaling (nMDS) plots. Total carbohydrates and total chlorophyll *a* were only possible on a few samples of *C*. *caespitosa* and for none of *A*. *calycularis* due to insufficient material and therefore excluded from the multivariate analyses. Additionally, individuals of either species lacking a complete set of data (respiration, calcification, biomass, total lipids, δ^15^N_w_, and lipid classes) were removed from the multivariate analysis to meet the assumptions of the analysis. This resulted in 1 of 24 *C*. *caespitosa* ramets and 16 of 39 *A*. *calycularis* ramets being excluded (4, 6, 5, and 8 individuals from the ambient–present day pH, ambient–low pH, vent–present day pH, and vent–low pH treatments, respectively). Vectors were added to nMDS plots using Pearson’s correlation analysis to determine the relative contribution of each variable to the distribution. Additionally, distance-based linear modeling (DistLM) was used to determine which physiological variables significantly affected differences in physiological profile. All multivariate analyses were performed using Primer V6 software [65].

To further investigate the data, each physiological variable was individually analyzed using univariate two-way ANOVA and post-hoc Tukey tests where site of origin (ambient vs vent) and treatment (present day pH vs. low pH) were fixed and fully crossed. Though multivariate analysis was completed on a subset of the samples that had all physiological measurements, univariate analyses were conducted using all available data for each variable. When univariate analyses were repeated using only the subset of the data used in the multivariate analyses, the results were the same. Therefore, we report the results of the univariate statistical analyses for all available data for each variable. Outliers identified with Cook’s distance of >0.5 were removed from individual univariate analyses. When assumptions of normality were not met (p < 0.05, Shapiro-Wilk test), data was transformed, or analyses were completed using the non-parametric Kruskal-Wallis test. Statistical results were considered significant at p ≤ 0.05.

## Results

**Fig 1:**
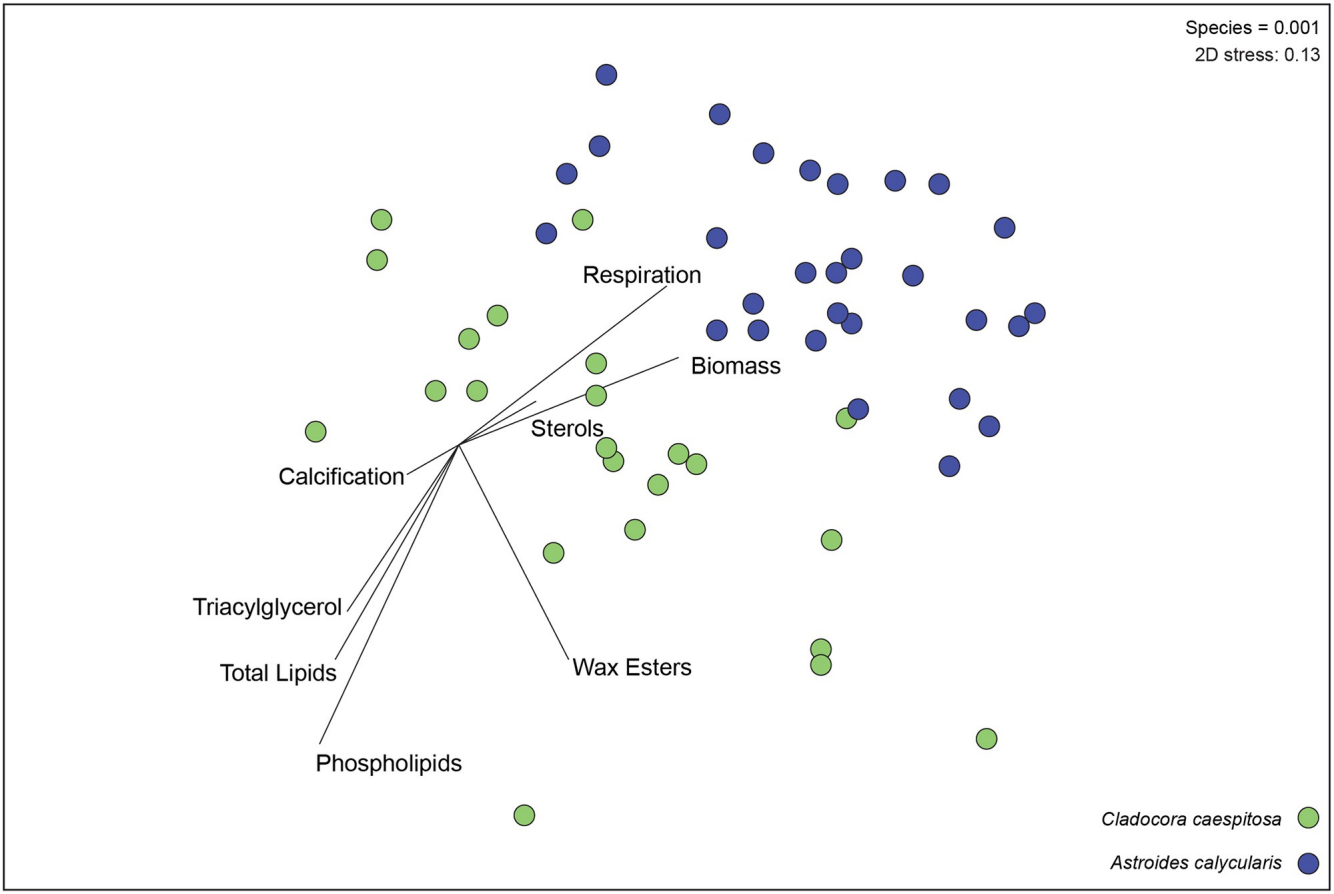
Multivariate NMDS plot of physiological profiles of *Cladocora caespitosa* and *Astroides calycularis*. Multivariate NMDS plot of physiological profiles using the variables calcification, respiration, photosynthesis, biomass, lipids, δ^15^N_h_ phospholipids, sterols, triacylglycerol, and wax esters, of *Cladocora caespitosa* (green) and *Astroides calycularis* (purple) individuals. Vectors indicate strength and direction of the relative contribution of each physiological variable to sample distribution as determined by Pearson’s correlation analysis. Corresponding PERMANOVA results indicate that the two species significantly differed from each other (p = 0.001).

Overall, the physiological profiles differed between *C*. *caespitosa* and *A*. *calycularis* (p-value = 0.001) with phospholipids, respiration, and total lipids driving 81% of the difference (Fig 1, S2 Table in S1 File).

### Cladocora caespitosa

**Fig 2:**
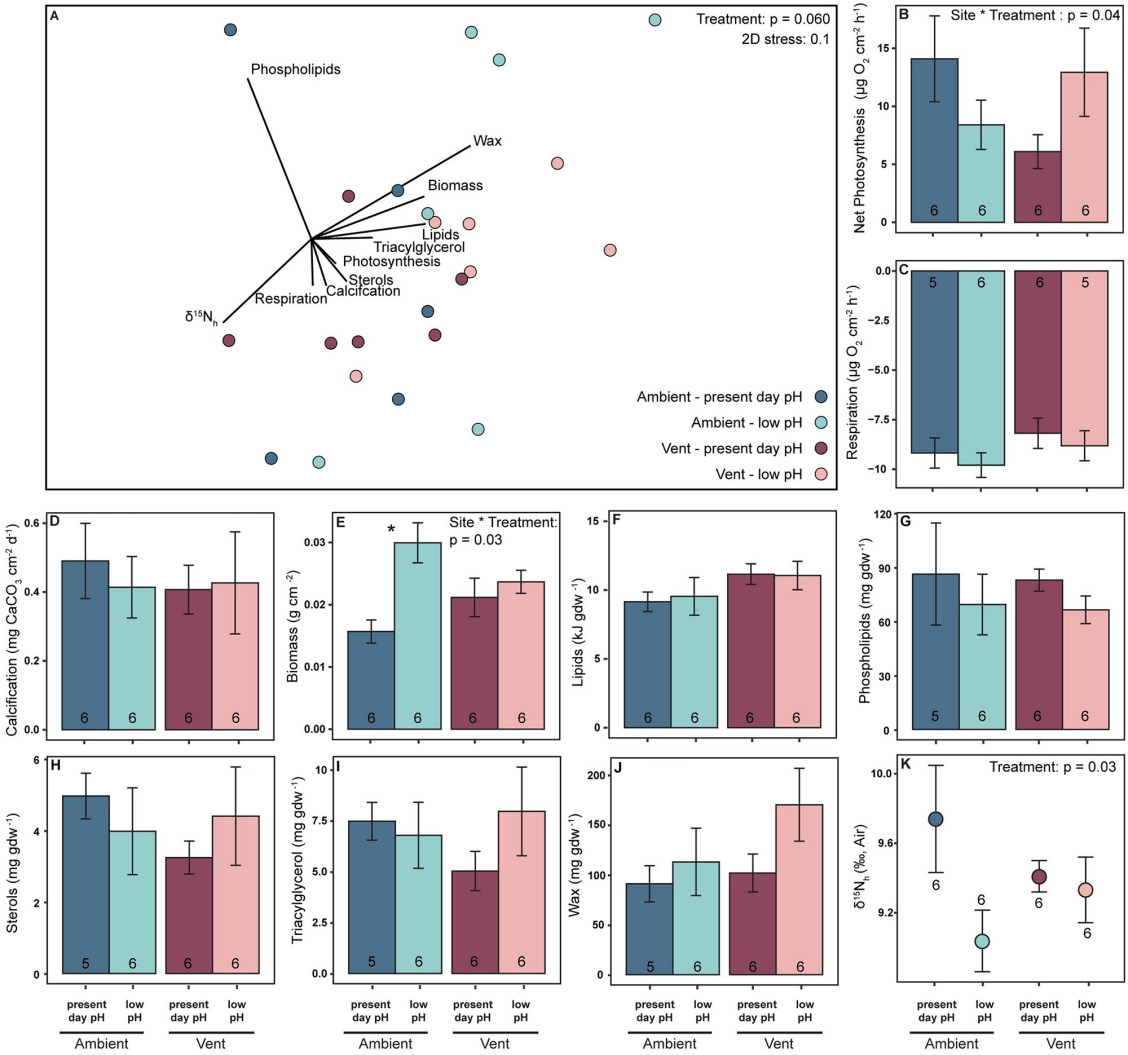
Multivariate NMDS and univariate results for *Cladocora caespitosa*. Multivariate (A) and univariate (B-K) results for *Cladocora caespitosa* corals sourced from the ambient site and experimentally reared at present day pH (dark blue, pH_T_ 8.08) and low pH (light blue, pH_T_ 7.72) and sourced from the vent site and experimentally reared at present day pH (dark pink, pH_T_ 8.08) and low pH (light pink, pH_T_ 7.72). A) Multivariate NMDS plot of physiological profiles (calcification, respiration, photosynthesis, biomass, lipids, δ^15^N_h_ phospholipids, sterols, triacylglycerols, and wax esters). Vectors indicate strength and direction of the relative contribution of each physiological variable to the sample distribution as determined by Pearson’s correlation analysis. Univariate results (B-K) represent the average (± 1 SE) of B) calcification, C) net photosynthesis, D) respiration, E) biomass, F), total lipids, G) phospholipids, H) sterols, I) triacylglycerols, J) wax esters, and K) δ^15^N_h_. Sample sizes for each average are indicated within each bar. Significant main effects from two-way ANOVA analyses are written in the top right of each panel. Asterisks denote significant differences due to experimental pH treatments between corals sourced from the same site. Statistical details in **S3-S6 Tables in** S1 File. Respiration, calcification, and photosynthesis data from Carbonne et al. (2021).

Physiological profiles of *C*. *caespitosa* did not differ by site of origin (Fig 2A, S3 Table in S1 File). However, there was a trend (p = 0.06) suggesting that the physiological profiles did differ between pH treatments, with 86% of the difference driven by phospholipids, wax esters, and δ^15^N_h_ (S4 Table in S1 File). Further analysis of each variable revealed that photosynthesis decreased in corals from the ambient–low pH treatment and increased in corals from the vent–low pH treatment relative to their present day pH counterparts (Fig 2B, S5 Table in S1 File). This result differed from results reported in Carbonne et al. [21] due to only a subset of samples being analyzed. Though not included in the multivariate analysis due to limited sample size, chlorophyll *a* was greater in corals collected from vent sites compared to ambient sites (S2 Fig, S5 Table in S1 File). As previously reported by Carbonne et al. [21], respiration and calcification did not differ between pH treatments, irrespective of the site of origin of the corals (Fig 2C and 2D; S5 Table in S1 File). Biomass increased in corals from the ambient site when exposed to experimentally low pH (Fig 2E, S5 Table in S1 File), while total lipids and carbohydrates were unaffected by treatment or site of origin (Fig 2F, S2 Fig, S5 Table in S1 File). The pH treatment and site of origin had no significant effect on the lipid class composition of *C*. *caespitosa* (Fig 2G–2J, S6 Table in S1 File). δ^15^N_h_ was significantly lower at low pH compared to present day pH (Fig 2K, S5 Table in S1 File), which was largely due to a decline in ambient-sourced corals. Additionally, within the present day pH treatment, δ^13^C_h_ was unaffected by site of origin while δ^13^C_h-e_ was significantly lower in corals collected from vent sites than ambient sites (S3 Fig, S11 Table in S1 File). SIBER results between host and algal endosymbionts in the ambient–present day pH treatment had 0% and 17% overlap for 40% SEA_c_ and 95% SEA_c_, respectively (S4A Fig in S1 File). In the vent -sourced corals in the ambient pH treatment, there was 0% overlap for both 40% and 95% SEA_c_ (S4B Fig in S1 File).

### Astroides calycularis

**Fig 3:**
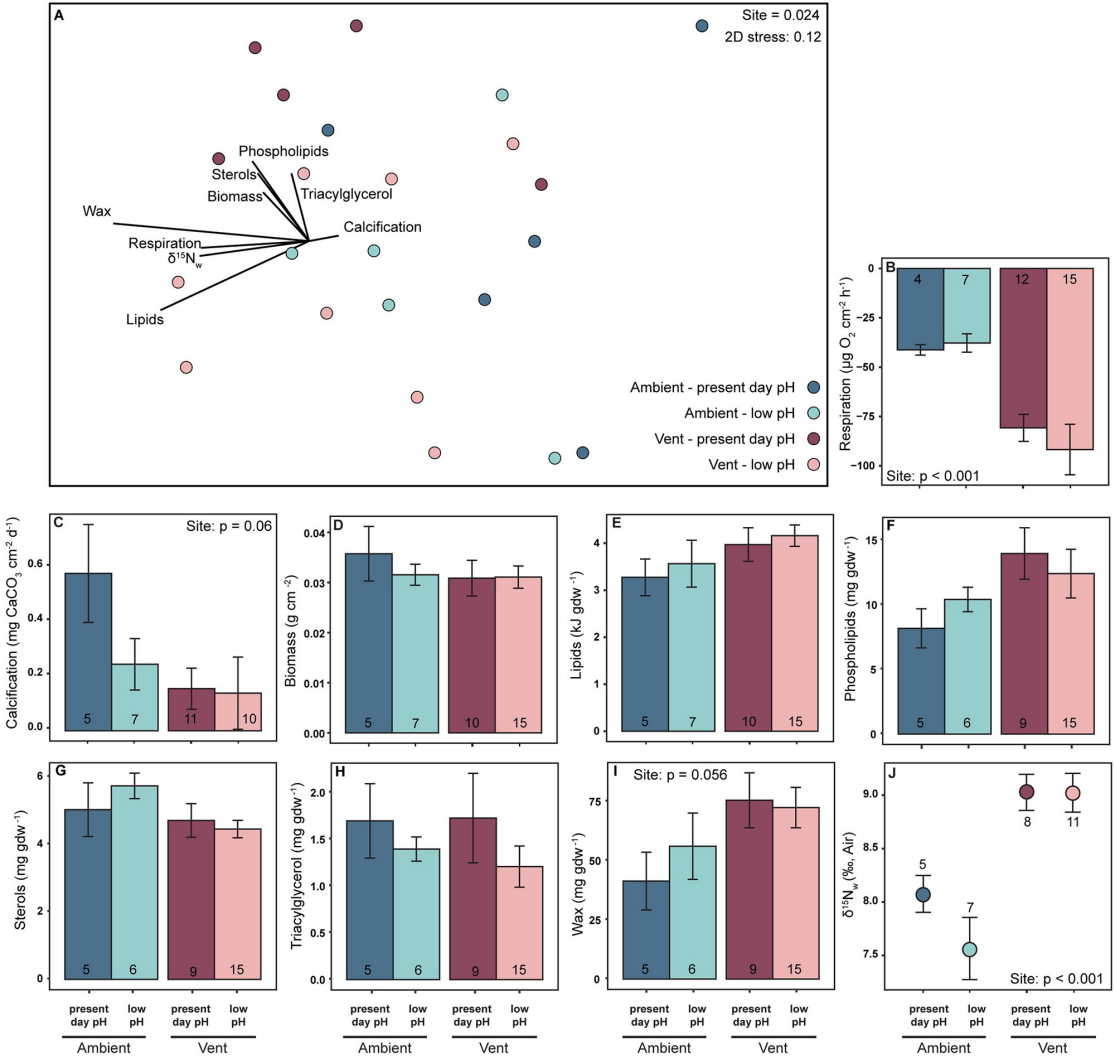
Multivariate NMDS and univariate results for *Astroides calycularis*. Multivariate (A) and univariate (B-J) results for *Astroides calycularis* corals sourced from the ambient site and experimentally reared at present day pH (dark blue, pH_T_ 8.08) and low pH (light blue, pH_T_ 7.72) and sourced from the vent site and experimentally reared at present day pH (dark pink, pH_T_ 8.08) and low pH (light pink, pH_T_ 7.72). A) Multivariate NMDS plot of physiological profiles (calcification, respiration, biomass, lipids, δ^15^N_w_, phospholipids, sterols, triacylglycerols, and wax esters). Vectors indicate strength and direction of the relative contribution of each physiological variable to the sample distribution as determined by Pearson’s correlation analysis. Univariate results (B-K) represent the average (± 1 SE) of B) calcification, C) respiration, D) biomass, E), total lipids, F) phospholipids, G) sterols, H) triacylglycerols, I) wax esters, and J) δ^15^N_w_. Sample sizes for each average are indicated within each bar. Significant main effects from two-way ANOVA analyses are written in the top right of each panel. Asterisks denote significant differences due to experimental pH treatments between corals sourced from the same site. Statistical details in **S7-S10 Tables in** S1 File. Respiration, calcification, and photosynthesis data from Carbonne et al. (2021).

Physiological profiles of *A*. *calycularis* from the vent site differed from those from the ambient site (Fig 3A, S7 Table in S1 File), with 77% of the difference being driven by wax esters, total lipids, and phospholipids (S8 Table in S1 File). As previously reported by Carbonne et al. [21], respiration was higher in corals sourced from vent sites than corals sourced from ambient sites (Fig 3B, S9 Table in S1 File). Calcification had a strong trend (p = 0.06) of being lower in vent-sourced corals than ambient-sourced corals irrespective of treatment (Fig 3C, S9 Table in S1 File). Corals in both treatments, irrespective of their site of origin, had similar biomass, total lipid, and lipid class concentrations (Fig 3D–3I, S9, S10 Tables in S1 File). Although there was a strong trend of higher wax esters in vent corals than ambient corals (p = 0.056) (Fig 3I, S10 Table in S1 File). δ^15^N_w_ was significantly higher in corals originally sourced from vent sites than from ambient sites (Fig 3J, S9 Table in S1 File).

## Discussion

In this study we quantified physiological differences in two species of corals–*C*. *caespitosa* and *A*. *calycularis*–collected from ambient sites and vent sites, that had been reared in present day pH (pH_T_ 8.08) or low pH (pH_T_ 7.72) seawater for six months. We evaluated if elevated heterotrophic capacity enabled Mediterranean corals to cope with low pH. As expected, the two species had overall different physiology (Fig 1). In addition, the overall physiological profiles of the symbiotic coral *C*. *caespitosa* differed between pH treatments, whereas the physiological profiles of the asymbiotic coral *A*. *calycularis* differed by site of origin (Figs 2 & 3).

### High heterotrophic capacity appears to underlie Mediterranean coral resilience to ocean acidification

Some corals exhibit trophic plasticity by altering their reliance on heterotrophy under stressful conditions to meet metabolic demand [31–33, 66]. Contrary to our hypothesis, we found no evidence that either *C*. *caespitosa* or *A*. *calycularis* increase their reliance on heterotrophy in response to experimentally induced decreases in pH. The decrease, or lack of change, in δ^15^N values and the maintenance of sterols in corals reared in low pH conditions sourced from both sites (Figs 2H, 2K, 3G and 3J) indicates assimilation of heterotrophic carbon did not increase in response to decreases in pH for both species. Instead, we find evidence of sustained heterotrophic capacity in vent-sourced corals and a decline in heterotrophic capacity in ambient-sourced corals when grown at low pH.

Decreases in δ^15^N in ambient-sourced corals of both species in the low pH treatment indicates a decrease, rather than increase, in heterotrophic assimilation into tissues (Figs 2K & 3J). However, energy reserve concentrations, including sterols, were similar in ambient corals at both experimental pH levels (Figs 2H & 3G). For *C*. *caespitosa* the apparent decoupling of heterotrophy and energy reserves may be misleading, as changes in biomass, total lipids, and lipid classes can lag behind changes in heterotrophy by several months [31, 45, 67, 68]. Additionally, because ambient-sourced corals maintained photosynthesis for the first month of the experiment [21], they may have been able to initially allocate fixed carbon to lipid synthesis and biomass accumulation [30, 67, 68] sufficiently to sustain energy reserves during the experiment. For *A*. *calycularis*, energy reserves may have been sustained by the trend of decreases in calcification at low pH (Fig 3C). For both species, the maintenance of energy reserves after six months may not represent the long-term effects of low pH on these corals. After six months, the isotopic value in both species suggests decreases in feeding, at low pH, when the corals were sourced from ambient sites (Figs 2K & 3J blue bars). We suspect if the experiment had run longer, the cumulative effect of reduced heterotrophic contributions would have resulted in decreases in calcification, energy reserves, and/or storage lipid classes (wax esters and triacylglycerols) in ambient–low pH corals. However, none of the trends or effects in the data indicate that coral sourced from vent sites would suffer any long-term declines.

Vent-sourced corals, of both species, maintained δ^15^N at low pH, indicating they were able to sustain their heterotrophic capacity in response to low pH. Maintenance of heterotrophy likely contributed to similar energy reserves in present day and low pH treatments, as up to 40% of lipids in healthy corals are synthetized from heterotrophically sourced carbon [30]. *C*. *caespitosa* collected from vents also increased photosynthesis at low pH (Fig 2B), most likely due to CO_2_ enrichment for photosynthesis [69], providing additional fixed carbon to the corals that could have been allocated for lipid synthesis [30], biomass accumulation, and/or calcification [29]. CO_2_ enrichment of photosynthesis was only evident in vent-sourced *C*. *caespitosa*, most likely due to previous long-term exposure and adaptation to low pH in the field [70, 71]. This is consistent with the finding of higher chlorophyll *a* concentrations in these corals (S2 Fig in S1 File). Additionally, within the present day pH treatment, vent-sourced *C*. *caespitosa* corals had lower δ^13^C_h-e_ and lower SIBER SEAc values than corals from the ambient site (S3, S4 Figs in S1 File), which indicates vent-sourced corals are more heterotrophic than ambient-sourced corals [64]. However, δ^13^C and SIBER were not included in the overall physiological profiles, which could understate the importance of collection site for *C*. *caespitosa*.

*A*. *calycularis* corals sourced from the vent site had higher δ^15^N_w_ than corals sourced from the ambient pH site, which was sustained at low pH (Fig 3J). This provides further evidence of greater feeding on the brine shrimp nauplii they were provided during the study (δ^15^N values of 12.44‰). Thus, energy reserves (i.e., total lipids and biomass) in vent-sourced *A*. *calycularis* appear to be sustained by high baseline heterotrophic capacity (Fig 3J). Additionally, increased feeding of vent *A*. *calycularis* could contribute to greater respiration demands allowing these corals to maintain calcification from increased concentrations of respired CO_2_ [21, 22, 72, 73]. The energy and nutrition obtained heterotrophically could have been used to meet metabolic demand, maintain positive rates of calcification, and/or increase concentrations of storage lipids (Fig 3I), as this has been observed in tropical corals [30, 31, 34, 43, 62].

Vent-collected *C*. *caespitosa* and *A*. *calycularis* showed maintenance of δ^15^N at low pH, suggesting that natural populations of these two species have a greater dependence on zooplankton than ambient-sourced populations, possibly due to local adaptation (Figs 2K & 3J). For *C*. *caespitosa*, zooplankton is a nutritious food that provides a dense source of essential nitrogen, phosphorous, amino acids, and other nutrients that cannot be obtained through photosynthesis [26]. Additionally, zooplankton is more nutritious than dissolved organic matter (DOM) or POM [74, 75], which is beneficial for both species. Although isotopic tissue values in this study do not allow us to discern the diet composition of these corals, the data suggests coral communities at vent sites may feed more on zooplankton than corals at ambient sites due to increased feeding effort (Figs 2K and 3J, S3 & S4 Figs in S1 File). Additionally, recent evidence suggests that heterotrophically acquired nitrogen may increase coral calcification [24, 25]. Therefore, increased zooplankton feeding capacity in CO_2_ vent-sourced corals of both species may allow these corals to maintain positive calcification and energy reserves in experimentally low pH conditions.

### Implications

For vent-sourced *C*. *caespitosa* at experimentally low pH, sustained heterotrophic feeding (Fig 2E) coupled with elevated photosynthesis rates (Fig 2B) could explain the maintenance of calcification and respiration [21], as well as biomass and lipids (Fig 2). Similarly for vent-sourced *A*. *calycularis*, elevated baseline heterotrophic capacity (Fig 3D and 3J) could explain higher respiration demands, maintenance of biomass and lipids, and the ability to maintain net positive calcification, even if at a lower rate than at ambient sites (Fig 3). Therefore, both species of corals sourced from vent sites show evidence of elevated assimilation of heterotrophic sources to their tissues. Direct field observations are needed to confirm this interpretation.

Overall, our study suggests selection for high baseline heterotrophic capacity on coral recruits that settle at CO_2_ vents. Low pH at vent sites could be a selective pressure that would contribute to recruit settlement [76, 77] and post-settlement success [78, 79]. Our data suggest low pH, vent sites may have selected for individuals that are able to maintain feeding at low pH, which in turn would support the maintenance of energy reserves and net positive calcification. Consistent with our findings is work by Teixidó et al [10] showing gene differentiation for calcification and pH regulation of *A*. *calycularis* between ambient and vent sites. However, further analyses on populations of *C*. *caespitosa* are needed to determine if CO_2_ ambient and vent populations have distinct gene expression that corresponds to environmental pH conditions.

Although ambient-sourced corals are tolerant of low pH in the short-term, our findings suggest these corals may not be able to acclimate to chronic low pH in the future oceans. However, the vent-sourced corals, of both species, appear to already be adapted for future ocean acidification conditions and use elevated heterotrophic capacity as a strategy to cope with long-term low pH conditions. Additional study is needed to determine how these corals will respond to the dual stress of ocean acidification and ocean warming. However, for *C*. *caespitosa* and *A*. *calycularis* to survive in the Mediterranean Sea with persistently low pH, CO_2_ vent populations will need to expand their ranges via sexual reproduction or through human intervention via restoration.

## Supporting information

S1 File (DOCX)

S1 Data (XLSX)
