# Supplementary material for: Elevated heterotrophic capacity as a strategy for Mediterranean corals to cope with low pH at CO2 vents
Source: PLoS One. 2024 Jul 30;19(7):e0306725. doi: 10.1371/journal.pone.0306725 (PMC11288460; doi:10.1371/journal.pone.0306725)
Supplement: S1 File — (DOCX) [file pone.0306725.s001.docx]

| Coral Collection | **Latitude and Longitude of collection site** | **Species collected and morphology** | **Collection date** | **Collection depth** | **Site pH_T_** | **Number of colonies collected** | **Symbiodiniaceae species** |
| --- | --- | --- | --- | --- | --- | --- | --- |
|  | Chiane Ambient: 40.718˚N, 13.965˚E | *Cladocora caespitosa* (Phaceloid, symbiotic) | May 7-8 2019 | 10m | 8.05 | 16 | *Breviolum psygmophilum* / *Philzoon sp.* |
|  | Chiane Vent: 40.718˚N, 13.960˚E | *Cladocora caespitosa* (Phaceloid, symbiotic) | May 7-8 2019 | 10m | 7.91 | 14 | *Breviolum psygmophilum* / *Philzoon sp.* |
|  | San Pancrazio: 40.701˚N, 13.954˚E | *Astroides calycularis* (Phaceloid, asymbiotic) | May 7-8 2019 | 1-2m | 7.97 | 14 | n/a |
|  | Grotta del Mago: 40.712˚N, 13.964˚E | *Astroides calycularis* (Phaceloid, asymbiotic) | May 7-8 2019 | 1-2m | 7.88 | 27 | n/a |
| Experimental Design | **Location of Experiment** | **Stress treatment period** | **Tank system type** | **Number of tanks per treatment (present day or low pH)** | **Number of coral genets per treatment** | **Acclimation prior to experiment** | **Number of recovery days post-stress** |
|  | Laboratoire Océanographique de Villefranche, France | 15 May – 2 December 2019 | Flow-through | 12 | 14-16 (*C. caespitosa*)  18-23 (*A. calyucularis*) | 1 week | n/a |
| Experimental Conditions | **Present day and low pH treatment temperature** | **Present day treatment pH_T_** | **Low pH treatment pH_T_** | **Duration at stress** | **Method of lowering pH** | **Salinity** |  |
|  | 22.7 ± 1.9˚C | 8.06 ± 0.06 | 7.73 ± 0.09 | 6 months | Bubbled CO_2_ gas | 37.8 ± 0.5 |  |
| Other experimental conditions | **Light conditions & cycle:** | **Flow rate**  **(cm s^-1^):** | **Tank turnover rate:** | **Seawater source and filtration:** | **Other abiotic variables** | **Coral feeding:** |  |
|  | Diurnal light cycle with maximum irradiance of 180 µmol photons m^-2^ s^-1^ (*A. calycularis* was shaded to prevent direct light) | Not measured | Not measured | Natural seawater pumped directly from Villefranche Bay | See Carbonne et al. 2021 for detailed carbonate chemistry. Nutrients and dissolved oxygen not measured | Fed 3x a week with freshly hatched brine shrimp |  |

**S1 Table**. **Collection and Experimental Metadata.** Metadata of coral collection and experimental treatments modified from Grottoli et al. 2021.

| Variable | P-value | Proportion Contribution |
| --- | --- | --- |
| Phospholipids | **0.001** | 0.32 |
| Respiration | **0.001** | 0.26 |
| Total Lipids | **0.001** | 0.23 |
| Biomass | **0.001** | 0.22 |
| Wax Esters | **0.001** | 0.19 |
| Triacylglycerol | **0.001** | 0.15 |
| Sterols | **0.013** | 0.08 |
| Calcification | 0.565 | 0.01 |

**S2 Table. DistLM for *Cladocora caespitosa* and *Astroides calycularis.*** Distance-based linear modelling (DistLM) results showing proportionate contribution of each variable to differences in physiological profile between *Cladocora caespitosa* and *Astroides calycularis*.

**S3 Table. PERMANOVA for *Cladocora caespitosa*.** Two-way PERMANOVA analysis to determine if the physiological profile (calcification, respiration, photosynthesis, biomass, lipids, ẟ^15^N_h_, phospholipids, sterols, triacylglycerol, and wax esters) of *Cladocora caespitosa* differs by site and treatment. Significant p-values are bolded.

| Species | PERMANOVA | Mean Squares | P-value |
| --- | --- | --- | --- |
| *Cladocora caespitosa* | Site | 3.79 | 0.728 |
|  | Treatment | 25.25 | 0.060 |
|  | Site*Treatment | 2.45 | 0.851 |
|  | Residuals | 8.79 |  |

**S4 Table. DistLM for *Cladocora caespitosa.*** Distance-based linear modelling (DistLM) results showing the proportionate contribution of each variable to differences in the physiological profiles of *Cladocora caespitosa* between sites and treatments.

| Variable | P-value | Proportion Contribution |
| --- | --- | --- |
| Wax Esters | **0.001** | 0.41 |
| Phospholipids | **0.001** | 0.26 |
| ẟ^15^N_h_ | **0.001** | 0.19 |
| Lipids | **0.006** | 0.15 |
| Biomass | **0.024** | 0.14 |
| Triacylglycerol | **0.048** | 0.13 |
| Sterols | 0.058 | 0.12 |
| Respiration | 0.567 | 0.03 |
| Calcification | 0.694 | 0.02 |
| Photosynthesis | 0.821 | 0.01 |

**S5 Table. Two-way ANOVA for *Cladocora caespitosa* univariate variables.** Two-way ANOVA analyses to determine if individual variables of *Cladocora caespitosa* differ by treatment (present day pH vs low pH) and collection site (ambient vs vent). Df = degrees of freedom, Mean Sq = mean squares. Significant p-values are bolded. Present day pH = pH_T_ of 8.08, low pH = pH_T_ of 7.72. Ambient = corals originally sourced form the ambient site, Vent = corals originally sourced from the vent site.

| Variable | Test | Effect | df | Mean Sq | F-value | P-value | post-hoc test |
| --- | --- | --- | --- | --- | --- | --- | --- |
| Calcification | Two-way ANOVA | Site | 1 | 0.08 | 1.068 | 0.747 |  |
|  |  | Treatment | 1 | 0.01 | 0.069 | 0.796 |  |
|  |  | Site*Treatment | 1 | 0.01 | 0.196 | 0.663 |  |
|  |  | Residuals | 20 | 0.07 |  |  |  |
| Photosynthesis | Two-way ANOVA | Site | 1 | 1.8*10^-5^ | 0.345 | 0.563 |  |
|  |  | Treatment | 1 | 1.9*10^-6^ | 0.038 | 0.847 |  |
|  |  | Site*Treatment | 1 | 2.3*10^-4^ | 4.502 | **0.046** |  |
|  |  | Residuals | 20 | 5.2*10^-5^ |  |  |  |
| Respiration | Two-way ANOVA | Site | 1 | 5.9*10^-6^ | 2.065 | 0.168 |  |
|  |  | Treatment | 1 | 2.1*10^-6^ | 0.728 | 0.405 |  |
|  |  | Site*Treatment | 1 | 3.0*10^-10^ | 0.000 | 0.991 |  |
|  |  | Residuals | 18 | 2.9*`0^-6^ |  |  |  |
| ẟ^15^N_h_ | Two-way ANOVA | Site | 1 | 0.043 | 0.187 | 0.671 |  |
|  |  | Treatment | 1 | 1.319 | 5.745 | **0.027** | Present day pH > Low pH |
|  |  | Site*Treatment | 1 | 0.310 | 1.349 | 0.259 |  |
|  |  | Residuals | 19 | 0.229 |  |  |  |
| Total Biomass | Two-way ANOVA | Site | 1 | 9.3*10^-7^ | 0.023 | 0.880 |  |
|  |  | Treatment | 1 | 2.1*10^-4^ | 10.501 | **0.004** |  |
|  |  | Site*Treatment | 1 | 1.4*10^-4^ | 5.153 | **0.034** |  |
|  |  | Residuals | 20 | 4.1*10^-5^ |  |  |  |
| Total Lipids | Two-way ANOVA | Site | 1 | 18.56 | 3.079 | 0.095 |  |
|  |  | Treatment | 1 | 0.13 | 0.021 | 0.885 |  |
|  |  | Site*Treatment | 1 | 0.37 | 0.061 | 0.808 |  |
|  |  | Residuals | 20 | 6.03 |  |  |  |
| Total chlorophyll *a* | Two-way ANOVA | Site | 1 | 1.3*10^6^ | 10.027 | **0.007** | Vent > Ambient |
|  |  | Treatment | 1 | 2.7*10^3^ | 0.216 | 0.649 |  |
|  |  | Site*Treatment | 1 | 3.6*10^3^ | 0.029 | 0.867 |  |
|  |  | Residuals | 13 | 1.3*10^5^ |  |  |  |
| Total Carbohydrates | Two-way ANOVA | Site | 1 | 5.6*10^-5^ | 1.331 | 0.263 |  |
|  |  | Treatment | 1 | 51.7*10^-5^ | 0.420 | 0.525 |  |
|  |  | Site*Treatment | 1 | 55 | 0.001 | 0.971 |  |
|  |  | Residuals | 18 | 4.2*10^-5^ |  |  |  |

**S6 Table. Two-way ANOVA for *Cladocora caespitosa* lipid classes.** Two-way ANOVA analyses to determine if lipid classes of *Cladocora caespitosa* differ by treatment (present day pH vs low pH) and collection site (ambient vs vent). Phospholipids and sterols were log transformed to meet assumptions of normality (Shapiro-Wilk > 0.05). Df = degrees of freedom, Mean Sq = mean squares. Significant p-values are bolded.

| Variable | Test | Effect | df | Mean Sq | F-value | P-value | post-hoc test |
| --- | --- | --- | --- | --- | --- | --- | --- |
| Phospholipids | Two-way ANOVA | Site | 1 | 0.06 | 0.299 | 0.591 |  |
|  |  | Treatment | 1 | 0.26 | 1.221 | 0.283 |  |
|  |  | Site*Treatment | 1 | 0.01 | 0.025 | 0.875 |  |
|  |  | Residuals | 19 | 0.22 |  |  |  |
| Sterols | Two-way ANOVA | Site | 1 | 0.16 | 0.452 | 0.509 |  |
|  |  | Treatment | 1 | 0.08 | 0.221 | 0.644 |  |
|  |  | Site*Treatment | 1 | 0.42 | 1.147 | 0.297 |  |
|  |  | Residuals | 19 | 0.36 |  |  |  |
| Triacylglycerol | Two-way ANOVA | Site | 1 | 1.7 3 | 0.123 | 0.73 |  |
|  |  | Treatment | 1 | 8.70 | 0.619 | 0.441 |  |
|  |  | Site*Treatment | 1 | 18.76 | 1.334 | 0.262 |  |
|  |  | Residuals | 19 | 14.1 |  |  |  |
| Wax Esters | Two-way ANOVA | Site | 1 | 6.35*`0^3^ | 1.418 | 0.249 |  |
|  |  | Treatment | 1 | 1.23*`0^4^ | 2.751 | 0.115 |  |
|  |  | Site*Treatment | 1 | 2.96*10^3^ | 0.660 | 0.427 |  |
|  |  | Residuals | 18 | 4.48*10^3^ |  |  |  |

**S7 Table. PERMANOVA for *Astroides calycularis.*** Two-way PERMANOVA analysis to determine if the physiological profile (calcification, respiration, biomass, lipids, ẟ^15^N_w_, phospholipids, sterols, triacylglycerol, and wax esters) of *Astroides calycularis* differs by site and treatment. Significant p-values are bolded.

| Species | PERMANOVA | Mean Squares | P-value |
| --- | --- | --- | --- |
| *Astroides calycularis* | Site | 18.54 | **0.024** |
|  | Treatment | 0.47 | 0.731 |
|  | Site*Treatment | 8.92 | 0.166 |
|  | Residuals | 5.53 |  |

**S8 Table. DistLM for *Astroides calycularis.*** Distance-based linear modelling (DistLM) results showing the proportionate contribution of each variable to differences in the physiological profiles of *Astroides calycularis*.

| Variable | P-value | Proportion Contribution |
| --- | --- | --- |
| Wax Esters | **0.001** | 0.36 |
| Lipids | **0.001** | 0.24 |
| Phospholipids | **0.006** | 0.17 |
| Respiration | **0.025** | 0.13 |
| ẟ^15^N_w_ | **0.044** | 0.12 |
| Sterols | 0.080 | 0.10 |
| Triacylglycerol | 0.133 | 0.08 |
| Biomass | 0.231 | 0.06 |
| Calcification | 0.471 | 0.04 |

**S9 Table. Two-way ANOVA for *Astroides calycularis* univariate variables.** Two-way ANOVA and Kruskal-Wallis analyses to determine if individual variables of *Astroides calycularis* differ by treatment (present day pH vs low pH) and collection site (ambient vs vent). Df = degrees of freedom, Mean Sq = mean squares. Significant p-values are bolded. Vent = corals originally sourced from vent site, Ambient = corals originally sourced from ambient sites.

| Variable | Test | Effect | df | Mean Sq | F-value | P-value | post-hoc test |
| --- | --- | --- | --- | --- | --- | --- | --- |
| Calcification | Two-way ANOVA | Site | 1 | 0.43 | 3.830 | 0.060 |  |
|  |  | Treatment | 1 | 0.14 | 1.230 | 0.276 |  |
|  |  | Site*Treatment | 1 | 0.19 | 1.691 | 0.204 |  |
|  |  | Residuals | 29 | 0.11 |  |  |  |
| Respiration | Kruskal-Wallis | Site | 1 |  |  | **<0.001** | Vent > Ambient |
|  |  | Treatment | 1 |  |  | 0.723 |  |
| ẟ^15^N_w_ | Two-way ANOVA | Site | 1 | 11.43 | 32.828 | **<0.001** | Vent > Ambient |
|  |  | Treatment | 1 | 0.30 | 0.866 | 0.360 |  |
|  |  | Site*Treatment | 1 | 0.46 | 1.327 | 0.259 |  |
|  |  | Residuals | 27 | 0.35 |  |  |  |
| Biomass | Two-way ANOVA | Site | 1 | 4.30*10^-5^ | 0.479 | 0.493 |  |
|  |  | Treatment | 1 | 1.36*10^-5^ | 0.151 | 0.699 |  |
|  |  | Site*Treatment | 1 | 3.79*10^-5^ | 0.423 | 0.520 |  |
|  |  | Residuals | 33 | 8.97*10^-5^ |  |  |  |
| Total Lipids | Two-way ANOVA | Site | 1 | 3.33 | 3.078 | 0.089 |  |
|  |  | Treatment | 1 | 0.45 | 0.021 | 0.526 |  |
|  |  | Site*Treatment | 1 | 0.02 | 0.061 | 0.892 |  |
|  |  | Residuals | 33 | 1.08 |  |  |  |

**S10 Table. Two-way ANOVA for *Astroides calycularis* lipid classes.** Two-way ANOVA and Kruskal-Wallis analysis to determine if lipid classes of *Astroides calycularis* differ by treatment (present day pH vs low pH) and collection site (ambient vs vent). Phospholipids were log-transformed and triacylglycerol was square-root transformed to meet assumptions of normality (Shapiro-Wilk > 0.05). Df = degrees of freedom, Mean Sq = mean squares. Significant p-values are bolded.

| Variable | Test | Effect | df | Mean Sq | F-value | P-value | post-hoc test |
| --- | --- | --- | --- | --- | --- | --- | --- |
| Phospholipids | Two-way ANOVA | Site | 1 | 0.51 | 2.143 | 0.1533 |  |
|  |  | Treatment | 1 | 2.4*10^-3^ | 0.010 | 0.9196 |  |
|  |  | Site*Treatment | 1 | 0.46 | 1.966 | 0.170 |  |
|  |  | Residuals | 31 | 0.24 |  |  |  |
| Sterols | Two-way ANOVA | Site | 1 | 5.59 | 3.575 | 0.068 |  |
|  |  | Treatment | 1 | 0.03 | 0.017 | 0.895 |  |
|  |  | Site*Treatment | 1 | 1.68 | 1.078 | 0.307 |  |
|  |  | Residuals | 31 | 1.56 |  |  |  |
| Triacylglycerol | Two-way ANOVA | Site | 1 | 0.13 | 0.6169 | 0.4382 |  |
|  |  | Treatment | 1 | 0.43 | 1.987 | 0.169 |  |
|  |  | Site*Treatment | 1 | 0.07 | 0.315 | 0.578 |  |
|  |  | Residuals | 31 | 0.21 |  |  |  |
| Wax Esters | Two-way ANOVA | Site | 1 | 4.37*10^3^ | 4.037 | 0.056 |  |
|  |  | Treatment | 1 | 62.50 | 0.057 | 0.812 |  |
|  |  | Site*Treatment | 1 | 576.20 | 0.532 | 0.471 |  |
|  |  | Residuals | 31 | 1.08*10^3^ |  |  |  |

**S11 Table. ANOVA for carbon isotopes of ambient pH treatment *Cladocora caespitosa.*** One-way ANOVA analysis to determine if individual variables of *C. caespitosa* in present day pH treatments differ by collection site. Df = degrees of freedom, Mean Sq = mean squares. Significant p-values are bolded.

| Variable | Test | Effect | df | Mean Sq | F-value | P-value |
| --- | --- | --- | --- | --- | --- | --- |
| ẟ^13^C_h_ | One-way ANOVA | Site | 1 | 1.21 | 3.93 | 0.076 |
|  |  | Residuals | 10 | 0.31 |  |  |
| ẟ^13^C_h-e_ | One-way ANOVA | Site | 1 | 4.60 | 6.95 | **0.025** |
|  |  | Residuals | 10 | 00.66 |  |  |

**
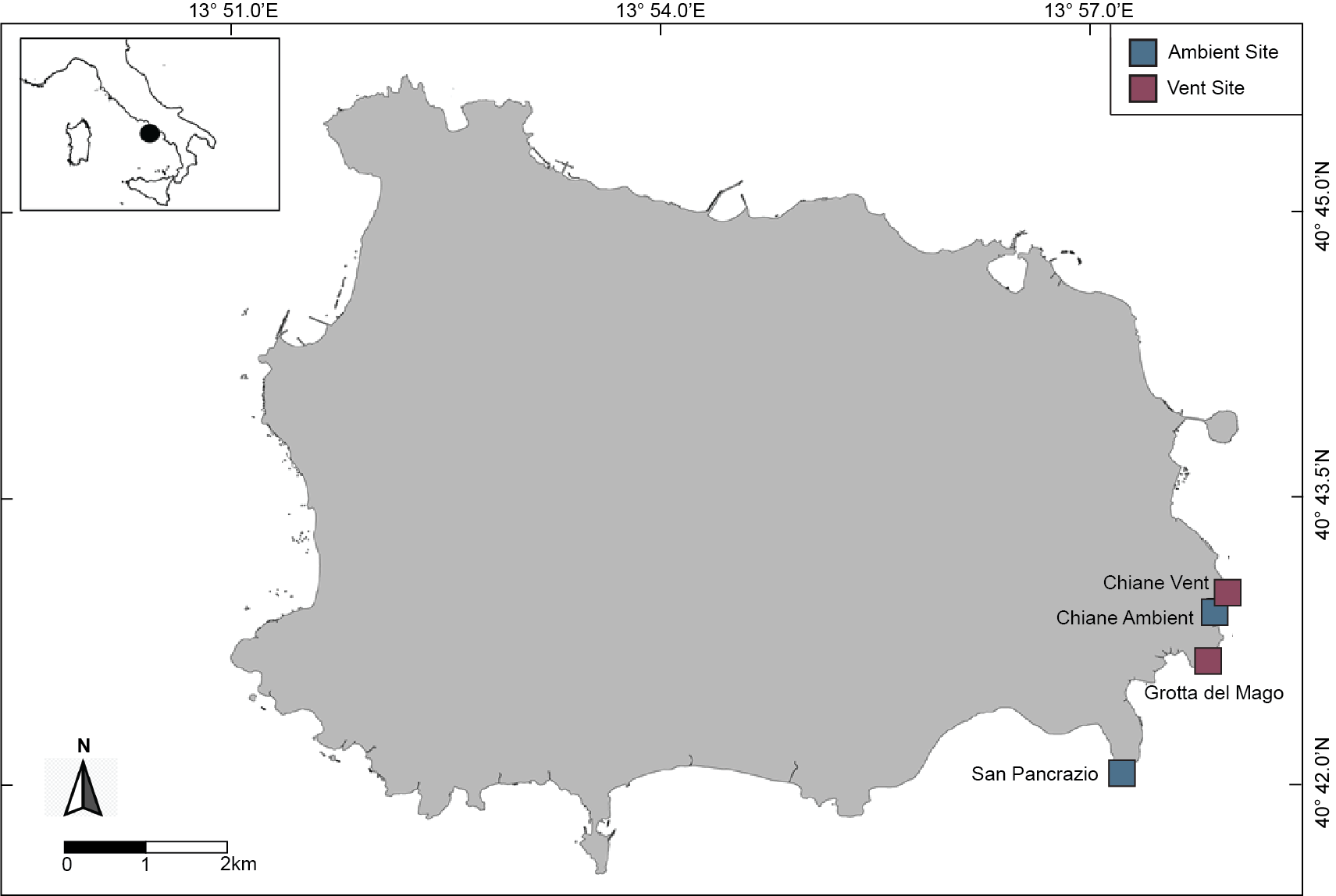
**

**S1 Figure. Map of collection sites.** Map showing collection sites of corals prior to experimental manipulation. *Cladocora caespitosa* corals were collected from the Chiane Vent (40.718˚N, 13.960˚E) and Chiane Ambient (40.718˚N, 13.965˚E) sites on 7-8 May 2019. *Astroides calycularis* corals were collected from the Grotta del Mago vent (40.711˚N, 13.694˚E) and ambient pH San Pancrazio (40.706˚N, 13.797˚E) sites on 7-8 May 2019.

**
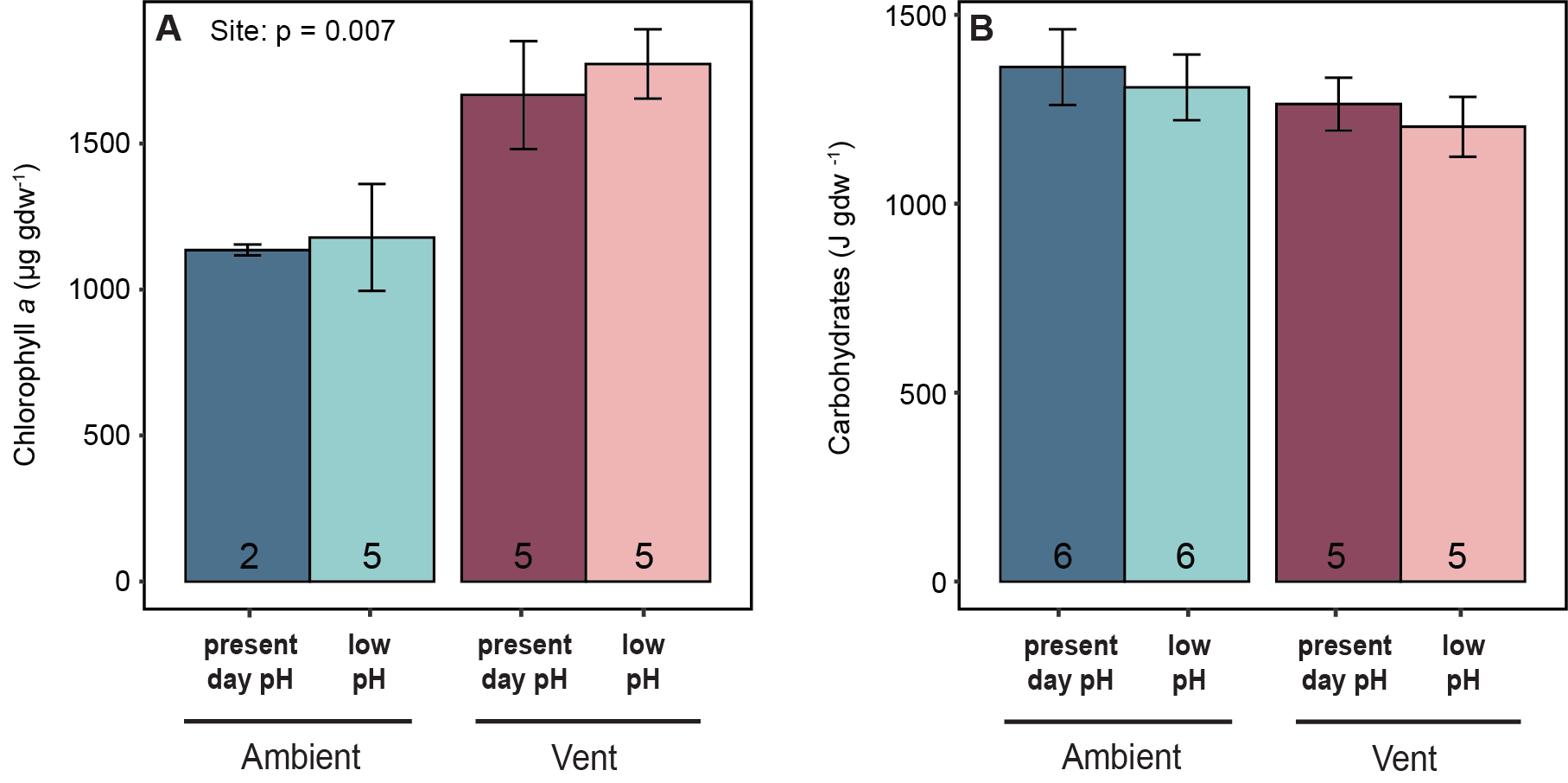
**

**S2 Figure. *Cladocora caespitsosa* chlorophyll and carbohydrate results.** Average (± 1 SE) A) total chlorophyll *a* and B) total carbohydrates in *Cladocora caespitosa* corals sourced from the ambient site and experimentally reared at present day pH (dark blue) or low pH (light blue) and sourced from the vent site and experimentally reared at present day pH (dark pink) and at low pH (light pink). Present day pH = pH_T_ of 8.08, low pH = pH_T_ of 7.72. Sample sizes for each average are indicated within each bar. Significant main effects from two-way ANOVA analyses are written in the top left of each panel. Asterisks denote significant differences between experimental pH treatments in corals sourced from the same site. Statistical details in **Table S5.**


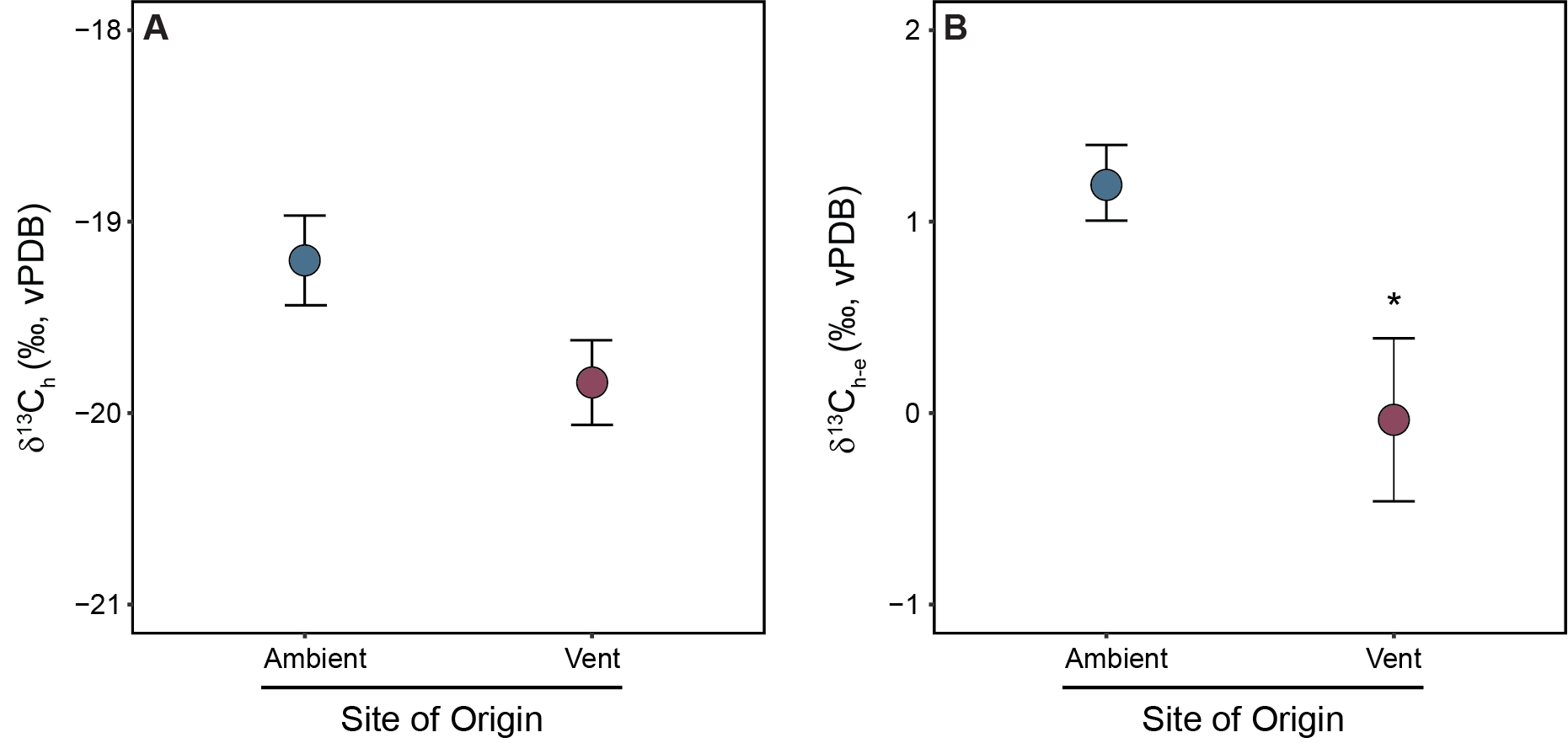


**S3 Figure. *Cladocora caespitosa* ẟ^13^C_h_ and ẟ^13^C_h-e_ of ambient pH treatment corals.** Average (± 1 SE) A) ẟ^13^C_h_ and B) ẟ^13^C_h-e_ in *Cladocora caespitosa* corals in the present day pH treatment (pH 8.08) sourced from the ambient site (dark blue) and the vent site (dark pink). * = significant differences between averages within a panel. Statistical details in **Table S11.**


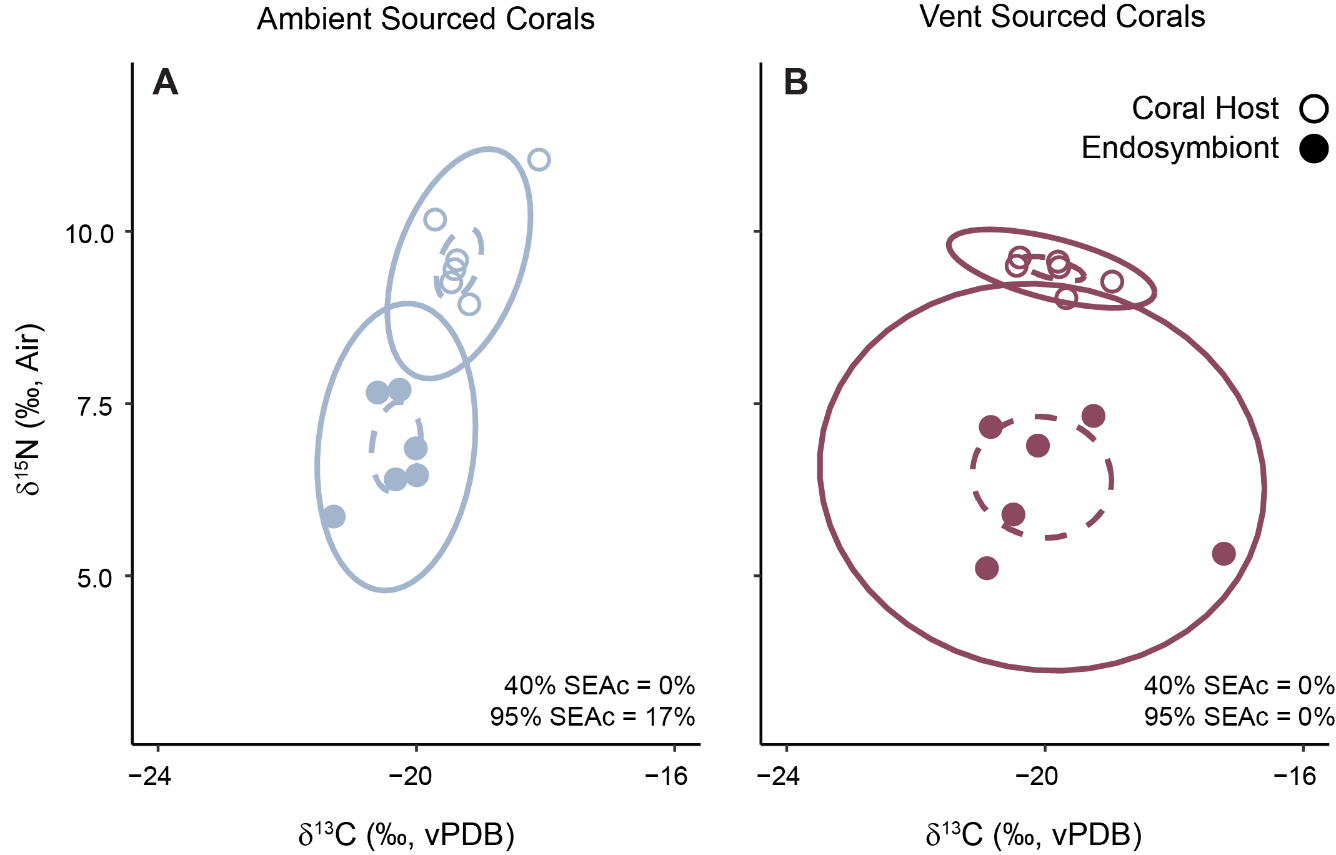


**S4 Figure. SIBER results of *Cladocora caespitosa* ambient pH treatment corals.** Results of SIBER analysis showing biplots of ẟ^13^C and ẟ^15^N for *Cladocora caespitosa* present day pH treatment corals sourced from A) Ambient sites (grey) and B) Vent sites (red). Dashed and solid line ellipses encompass 40% and 95% of the variability in host (open circles) and algal endosymbiont (closed circles) groups, respectively. Degree of overlap for both the 40% and 95% ellipses were calculated from the standard ellipse area corrected for sample size (SEAc) is a measure of the potential for resource sharing between the coral host and algal endosymbionts. Generally, heterotrophy increases as SEAc values decrease (Conti-Jerpe et al 2020). A similar analysis for the low pH treatment corals was not possible, as the CO_2_ gas used to create the low pH seawater imparted an isotopic signature onto the corals in those treatments, confounding the biological ẟ^13^C signature.
